# IRC data for a mechanistic route starting with H_2_O adsorption and finishing with H_2_ desorption from graphene

**DOI:** 10.1016/j.dib.2020.105362

**Published:** 2020-02-29

**Authors:** Andrea Oyarzún, Ximena García, Ljubisa Radovic

**Affiliations:** a Universidad de Magallanes, Chile; b Universidad de Concepción, Chile; c The Pennsylvania State University, USA

**Keywords:** Water rotation, Water adsorption, Hydrogen desorption, Water gasification, Reaction mechanism, *ab initio*

## Abstract

Intrinsic reaction coordinate (IRC) data regarding the interactions of water with a carbene-like active site located at the edge of a polyaromatic hydrocarbon [1–3] has been obtained using density functional theory (DFT) and the 6–31g(d) basis set as implemented in the Gaussian 16 software [4]. The data is presented as two videos (frontal and lateral mechanism views) combining four consecutive IRC calculations corresponding to the four different transition states presented on “https://doi.org/10.1016/j.carbon.2020.01.011” [3] (Figure 6, side approach). These videos provide powerful insights on two key aspects: a) the rotational process that occurs during water adsorption and b) the hydrogen gas desorption process during water gasification of carbons.

**Table:** Specifications Table

Subject	Physical and Theoretical Chemistry
Specific subject area	Mechanism data regarding the interactions of water with a carbene-like active site located at the edge of a polyaromatic hydrocarbon (during water gasification)
Type of data	Video
How data were acquired	Gaussian 16 program package (IRC method for transition states)
Data format	Raw Video-Compiled
Parameters for data collection	Forward and backward IRC calculations for transition states using DFT with the B3LYP functional and the 6–31g(d) basis set. The temperature is 298 K and the pressure is 1 atm. Forces are calculated at the first point and recalculated every 5 steps. The step size used is the default.
Description of data collection	The IRC calculation performs an optimization in the direction of the negative vibrational frequency of the transition state. Data was calculated for as much steps as possible with a maximum of 30 on each direction. A video was generated using GaussView for each IRC. iMovie was then used for producing the final mechanism video.
Data accessibility	Repository name: Mendeley Data Data identification number: 10.17632/6nwmtzcfxr.1 Direct URL to data: https://data.mendeley.com/datasets/6nwmtzcfxr.1
Related research article	Oyarzún, A., García-Carmona, X., and Radovic, L. *Kinetics of oxygen transfer reactions on the graphene surface. Part II. H2O vs CO2*. Carbon. Being submitted together. https://doi.org/10.1016/j.carbon.2020.01.011

**Table:** 

**Value of the Data** • Data presents *ab initio* evidence of the water adsorption phenomena, which from the molecular point of view presents a rotation of the molecule as it approaches to the active site. Also, an H_2_ desorption path for water gasification is presented, which requires the combination of two specific contiguous sites. • This data is important for researchers studying water reactions where slow rates are seen, and where slow diffusion mechanisms might be assumed under the lack of other evidence. As an example, this might be relevant for researchers studying hydration of macromolecules such as proteins [5]. • Data can be used as an *ab initio* evidence of special rotational features of the water molecule and also with educational purposes. • The data has also an educational value, because it presents a full reaction mechanism for water gasification on a single carbene-like active site from the adsorption step to the desorption of H_2_ as the first product.

## 1 Data

Two videos regarding the interactions of water with a carbene-like active site located at the edge of a polyaromatic hydrocarbon [1,2] are presented [6], H2OCarbeneSide_front.mp4 and H2OCarbeneSide.mp4. The first one shows the frontal view of the IRC sequence for the reaction mechanism of a water molecule with a carbene-like active site from the adsorption process to the H_2_ desorption process. The second one presents the lateral view of the same process. Both videos share the same timing and are labelled as described in Ref. [3]. Reverse IRC parts are indicated as uphill processes, forward IRC paths are indicated as downhill processes, transition states are labelled with their corresponding activation energy in kcal/mol and frozen for 3 seconds in order to capture there characteristics; finally, intermediate structures are also labelled with a name and their relative energy with respect to the reactants.

## 2 Experimental design, materials, and methods

Forward and backward IRC calculations for four different transition states using DFT with the B3LYP functional and the 6–31g(d) basis set were calculated [4]. The temperature and pressure are 298 K and 1 atm. Forces are calculated at the first point and recalculated every five steps. The step size was the software default size.

The resulting IRC calculations correspond to an optimization in the direction of the negative vibrational frequency of the transition state. Data was calculated for as many steps as possible with a maximum of 30 on each direction. A video was generated using GaussView for each IRC. iMovie was then used for producing the final mechanism video. Labelling of the structures to match Figure 6 (side approach) of [3] is also added.

## Appendix A Supplementary data

Multimedia component 1 Multimedia component1

## References

[1] Oyarzún A.M. (2015). Thermochemistry and Kinetics of Oxygen Transfer Reactions on the Graphene Surface: A Comparison of Interactions between NO, O2, H2O and CO2 and Carbene-like Active Sites.

[2] Oyarzún-Aravena A., Salgado-Casanova A., Radovic L., García-Carmona X. (2018). Thermodynamics and kinetics of graphene and H2O/CO2 reactions: insights of multiple-site edge effects. Carbon 2018: the World Conference on Carbon.

[3] Oyarzún A., García-Carmona X., Radovic L. (2020). Kinetics of Oxygen Transfer Reactions on the Graphene Surface. Part II. H2O vs CO2.

[4] Frisch M.J., Trucks G.W., Schlegel H.B., Scuseria G.E., Robb M.A., Cheeseman J.R. (2016). Gaussian 16 Revision A.03.

[5] Laage D., Hynes J.T. (2006). A molecular jump mechanism of water reorientation. Science.

[6] Oyarzún A.M., García-Carmona X., Radovic L.R. (2020). IRC data for a mechanistic route starting with H2O adsorption and finishing with H2 desorption from graphene. Mendeley Data.

